# Cytocompatible FRET Assembly of CdTe@GSH Quantum Dots and Au@BSA Nanoclusters: A Novel Ratiometric Strategy for Dopamine Detection

**DOI:** 10.3390/molecules30214169

**Published:** 2025-10-23

**Authors:** Arturo Iván Pavón-Hernández, Doris Ramírez-Herrera, Eustolia Rodríguez-Velázquez, Manuel Alatorre-Meda, Miguel Ramos-Heredia, Antonio Tirado-Guízar, Georgina Pina-Luis

**Affiliations:** 1 Centro de Graduados e Investigación en Química, Tecnológico Nacional de México/Instituto Tecnológico de Tijuana, Tijuana 22510, BC, Mexico; arturo.ph@tijuana.tecnm.mx (A.I.P.-H.); doris_e_777@hotmail.com (D.R.-H.); miguel.ramos193@tectijuana.edu.mx (M.R.-H.); antonio.tirado@tectijuana.edu.mx (A.T.-G.); 2 Facultad de Odontología, Universidad Autónoma de Baja California, Parque Industrial Internacional, Tijuana 22390, BC, Mexico; eustolia.rodriguez@uabc.edu.mx; 3 Tecnológico Nacional de México/I. T. Tijuana, Centro de Graduados e Investigación en Química-Grupo de Biomateriales y Nanomedicina, Blvd. Alberto Limón Padilla S/N, Tijuana 22510, BC, Mexico; 4 SECIHTI-Tecnológico Nacional de México/I. T. Tijuana, Centro de Graduados e Investigación en Química-Grupo de Biomateriales y Nanomedicina, Blvd. Alberto Limón Padilla S/N, Tijuana 22510, BC, Mexico; manuel.alatorre@tectijuana.edu.mx

**Keywords:** Förster resonance energy transfer, dopamine nanosensor, quantum dots, metallic nanocluster

## Abstract

This study presents a novel ratiometric fluorescent sensor based on Förster resonance energy transfer (FRET) between glutathione (GSH)-coated CdTe quantum dots (CdTe/GSH QDs) and bovine serum albumin (BSA)-coated Au nanoclusters (AuNCs/BSA) for dopamine (DA) detection. The nanoparticles were characterized using transmission electron microscopy (TEM), zeta potential measurements, Fourier transform infrared (FTIR) spectroscopy, UV-Vis absorption and fluorescence spectroscopy. Key FRET parameters, including energy transfer efficiency (*E*), donor–acceptor distance (*r*), Förster distance (*R*_0_), and the overlap integral (*J*), were determined. The interactions between the CdTe/GSH-AuNCs/BSA conjugate and DA were investigated, revealing a dual mechanism of QDs fluorescence quenching that involves both energy and electron transfer. The average lifetime values and spectral profiles of CdTe/GSH QDs, both in the absence and presence of DA, suggest a dynamic fluorescence quenching process. The variation in the ratiometric signal with increasing DA concentration demonstrated a linear response within the range of 0–250 µM, with a correlation coefficient of 0.9963 and a detection limit of 6.9 nM. This proposed nanosensor exhibited selectivity against potential interfering substances, including urea, glucose, BSA, GSH, citric acid, and metal ions such as Na^+^ and Ca^2+^. The conjugate also demonstrates excellent cytocompatibility and enhances cell proliferation in HeLa epithelial cells, making it suitable for biological applications. It was successfully employed for DA detection in urine samples, achieving recoveries ranging from 99.1% to 104.2%. The sensor is highly sensitive, selective, rapid, and cost-effective, representing a promising alternative for DA detection across various sample types.

## 1. Introduction

Dopamine (3,4-dihydroxyphenyl ethylamine, DA) is a crucial catecholamine neurotransmitter in the mammalian central nervous system. It also plays a significant role in the hormonal, renal, and cardiovascular systems [1]. DA dysfunction is associated with several neurological disorders, including schizophrenia, Parkinson’s disease, and anorexia [2,3,4,5]. Therefore, sensitive and precise determination of DA is essential for diagnosing and monitoring neurological diseases. Various strategies have been developed for DA detection, including chromatography [6,7,8], electrochemical methods [9,10,11,12], capillary electrophoresis [13], fluorescence [14], and kinetic-spectrophotometric techniques [15,16]. However, many of these methods do not meet the requirements for sensitivity, selectivity, speed of analysis, and cost-effectiveness. Although High-Performance Liquid-Chromatography (HPLC) offers high resolution for DA detection, it remains costly and often requires complex and time-consuming procedures. While electrochemical methods are more straightforward and economical, their results can be affected by other electroactive species, such as uric acid (UA) and ascorbic acid (AA), whose oxidation potentials are close to that of DA, leading to interference in the voltametric response [17]. Consequently, the development of selective and simple methods for DA detection in biological matrices, without the need for derivatization or separation steps, is highly desirable.

The use of fluorescence spectroscopy for DA detection has garnered increasing interest due to its high sensitivity and ease of application in biological samples. Recent advances in materials science and nanotechnology have led to the development of various nanosensors based on fluorescence detection. Notable examples include polypyrrole/graphene quantum dot hybrids [18], graphene oxide [19], graphene quantum dots [20], thiolate-protected gold nanoclusters [21], phosphate-modified TiO_2_ nanoparticles [22], and adenosine-capped quantum dots [23], among others, which have been employed for DA detection. Although these methods demonstrate good performance in DA determination, their recognition mechanism typically relies on fluorescence quenching.

Förster resonance energy transfer (FRET) has gained significant attention as a superior technique to simple fluorescence, especially in the study of interactions between emerging nanomaterials as a powerful and promising sensory tool for valuable applications in clinical diagnostics, biological assays, and environmental testing. FRET involves non-radiative energy transfer from an excited donor (typically a fluorophore) to a nearby ground-state acceptor through dipole–dipole interactions. An efficient FRET process typically occurs when there is significant overlap between the donor’s emission spectrum and the acceptor’s absorption spectrum, and when the donor and acceptor are within close proximity (1–10 nm). Nanocrystalline semiconductor quantum dots (QDs) are among the most intriguing new materials due to their unique optical and spectroscopic properties, making them ideal fluorescent labels for sensing and bioimaging applications [24,25,26]. QDs have proven to be highly effective FRET donors, thanks to their broad absorption spectrum, narrow and symmetric emission, and chemically accessible surface. FRET assemblies between QDs and various acceptors, such as dyes, gold nanoparticles (AuNPs), and QDs of different sizes, have been extensively studied. However, organic dyes often suffer from low photostability, while AuNPs of varying sizes exhibit a broadening of the surface plasmon resonance peaks as their diameter increases, particularly at longer wavelengths (above 650 nm). Compared to organic dyes or gold nanoparticles (AuNPs), metal nanoclusters (NCs) have garnered considerable interest due to their precisely defined optical properties. Metal NCs are a novel type of luminescent nanomaterial and are considered inorganic-organic hybrid compounds. They consist of a few to several tens of atoms, with diameters smaller than 3 nm, positioning them between atomic and plasmonic metal nanoparticles [27]. Unlike plasmonic nanoparticles, which exhibit semi-continuous electronic structures, metal NCs feature discrete electronic states due to quantum confinement effects. Distinctly, gold NCs do not display surface plasmon resonance but instead exhibit fluorescence in the visible-to-near-infrared (NIR) range. Metal NCs with diameters below 3 nm possess unique properties such as fluorescence, redox behavior, and magnetism, making them comparable to molecules in certain aspects [28,29]. Additionally, metal NCs are characterized by low toxicity and high photostability [30].

**Scheme 1:**
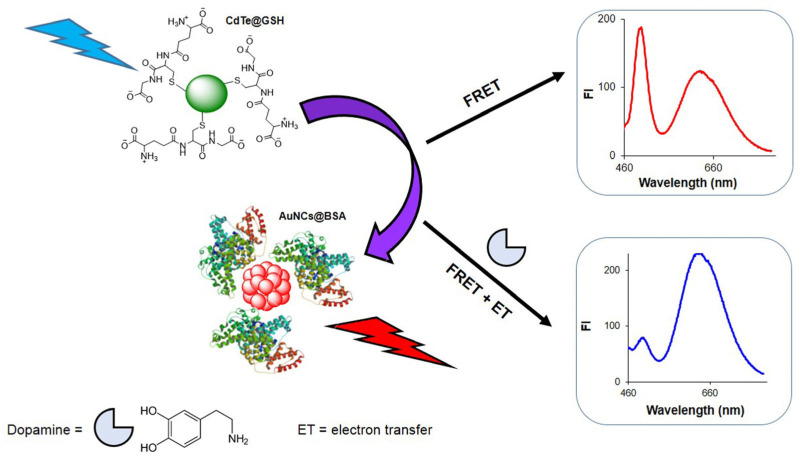
Schematic illustration of the sensor mechanism for DA detection.

Among the various properties of NCs, their fluorescence is particularly appealing as it offers a novel approach for developing photoluminescent sensors for biological applications. Particularly, FRET systems utilizing NCs have been relatively underexplored. In this study, we introduce a new nanosensor based on FRET assemblies involving glutathione (GSH) coated-CdTe quantum dots (CdTe/GSH QDs) as donors and bovine serum albumin (BSA) coated-Au nanoclusters (AuNCs/BSA) as acceptors. The DA detection mechanism of this nanosensor is shown in Scheme 1. In the presence of DA, the fluorescent signal of the donor decreases while the signal of the acceptor increases, leading to an enhanced fluorescence ratiometric signal consistent with a FRET behavior. Subsequent DA additions, lead to complete quenching, while the acceptor’s signal does not increase further, indicating a significant non-FRET contribution, attributed to electron transfer (ET). This ratiometric nanosensor demonstrates linearity within the concentration range of 0–250 µM, with a correlation coefficient of 0.9963 and a detection limit of 6.9 nM. The proposed method shows selectivity against other potential interfering substances and demonstrates excellent cytocompatibility. Furthermore, the FRET nanosensor was successfully applied to DA determination in urine samples, achieving excellent recovery rates ranging from 99.1% to 104.2%.

## 2. Results and Discussion

### 2.1. Synthesis and Characterization of CdTe/GSH QDs and AuNCs/BSA

#### 2.1.1. Synthesis and Spectral Characteristics of CdTe/GSH QDs

CdTe QDs coated with GSH were synthesized at varying reaction times. The typical progression of the UV-Vis absorption and fluorescence spectra of GSH-capped CdTe QDs over time is shown in Figure S1a (Supplementary Information). As seen in the figure, the spectra of the QDs shift to longer wavelengths as reaction time increases from 0 to 120 min. Additionally, narrow and symmetric bands with full width at half maximum (FWHM) between 33 and 67 nm confirm the presence of monodisperse and homogeneous CdTe/GSH QDs. Figure S1b shows the CdTe/GSH QDs under UV light.

**Figure 1:**
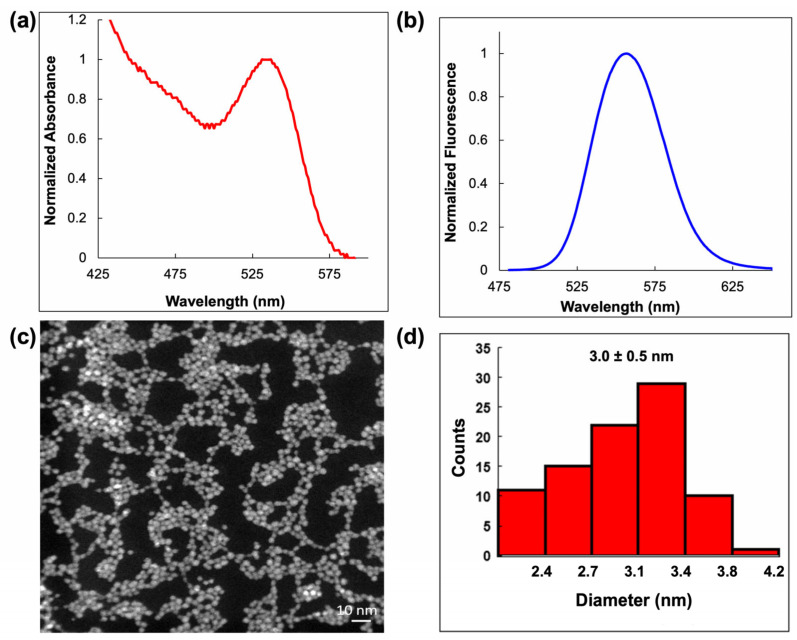
(**a**) Absorption and (**b**) fluorescence emission spectra of CdTe/GSH QDs, (**c**) HAADF image from CdTe/GSH QDs and (**d**) histogram.

The diameter of the CdTe/GSH QDs was calculated using the regression reported by Peng [31]. The main characteristics of the QDs obtained are listed in Table S1. Figure 1a,b show the UV-Vis absorption and fluorescence spectra of CdTe/GSH obtained after 30 min of reaction time. The absorption spectrum displays an excitonic peak at 535 nm and a corresponding emission band at 557 nm, with a FWHM of 52 nm. This absorption band is attributed to the transition of an electron from the highest energy state of the valence band to the lowest energy state of the conduction band (1S(h) → 1S(e)) in the CdTe/GSH QDs. These results suggest a nanoparticle diameter of 3.0 nm, consistent with the emission maximum at 557 nm. (Figure 1c,d) present the TEM image and corresponding histogram of CdTe/GSH, showing a uniform composition with a diameter of approximately 3.0 ± 0.5 nm, which aligns with the results obtained using Peng’s regression.

To confirm the incorporation of GSH on the surface of the QDs, Fourier Transform Infrared Spectroscopy (FT-IR) spectra of GSH and CdTe/GSH QDs were obtained. As shown in Figure S2, both spectra display a broad, strong absorption in the 3600–2700 cm^−1^ region, attributed to overlapping O-H and NH_3^+^_ stretching bands. Additionally, a weak, asymmetrical NH_3^+^_ bending band appears near 1610–1590 cm^−1^, along with a stronger symmetrical NH_3^+^_ bending band at 1550–1481 cm^−1^. The characteristic bands of carboxylate ions are observed near 1600–1590 cm^−1^ (asymmetrical) and more weakly around 1400 cm^−1^ (symmetrical), corresponding to carboxylate stretching. Notably, the carboxylate stretching overlaps with NH_3^+^_ bending bands from primary and secondary amines. The most significant observation is the disappearance of the SH stretching band at 2550 cm^−1^, confirming the successful incorporation of GSH on the QD surface.

#### 2.1.2. Synthesis and Spectral Characteristics of AuNCs/BSA

**Figure 2:**
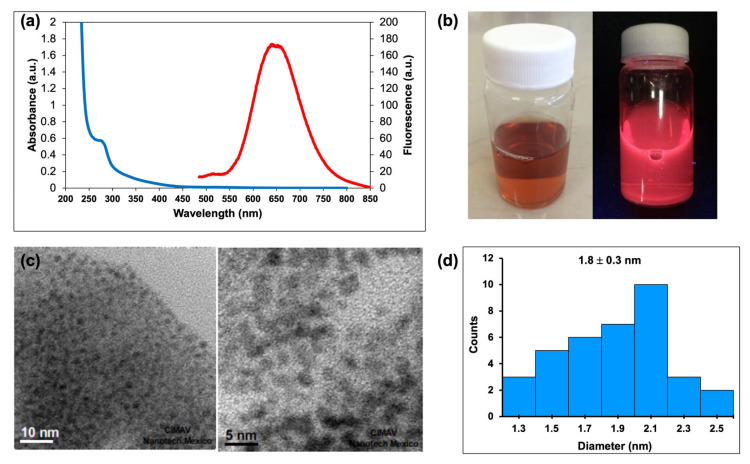
(**a**) Absorption (blue line) and fluorescence emission (red line) spectra of AuNCs/BSA (**b**) AuNCs/BSA under ambient light (left) and UV light (right) (**c**) STEM image from AuNCs/BSA and (**d**) histogram.

The synthesis of AuNCs/BSA utilizes BSA for its benign nature and dual role as both a reducing and capping agent in an aqueous medium, facilitating the nucleation and growth of AuNCs [32]. In BSA, the cysteine and histidine residues coordinate with Au ions, while tyrosine residues act as reducing agents to convert these ions into AuNCs, which are then stabilized by BSA. Tyrosine’s reducing ability is enhanced at pH levels above 10, which exceeds its pKa. The optical characteristics of the aqueous AuNCs@BSA solution were examined using UV-Vis absorption and molecular fluorescence spectroscopy as shown in Figure 2a. In a well-dispersed 100 nM aqueous solution, the nanoclusters exhibited a strong fluorescence band with a peak emission at 642 nm. The emission band has been attributed to intra-band transitions of free electrons in the AuNCs. The AuNCs@BSA also exhibit a well-defined excitation band with a maximum at 280 nm and a quantum yield of 8%. Figure 2b shows a solution of AuNCs/BSA under ambient light (left) and UV light (right). Figure 2c presents the bright field TEM image of the as-prepared BSA-capped AuNCs, and Figure 2d displays the corresponding histogram. The images reveal a spherical morphology, good dispersion, and a narrow size distribution of the nanoclusters, with an average size of 1.8 ± 0.3 nm.

### 2.2. Optical Stability of CdTe/GSH and AuNCs/BSA

The nanoparticles were also exposed to UV irradiation to evaluate their photostability. As shown in Figure S3, both nanoparticles demonstrate excellent photostability; after 60 min of irradiation at 365 nm, only a 10% decrease in fluorescence was observed for CdTe/GSH QDs and a 15% decrease for AuNCs/BSA. This stability makes them suitable for applications that require continuous irradiation. These findings are noteworthy, as most organic dyes are highly sensitive to UV light over similar durations.

### 2.3. Study of FRET Process Between CdTe/GSH QDs and AuNCs/BSA Assemblies

#### Selection of Optimal Conditions of the FRET Process

**Figure 3:**
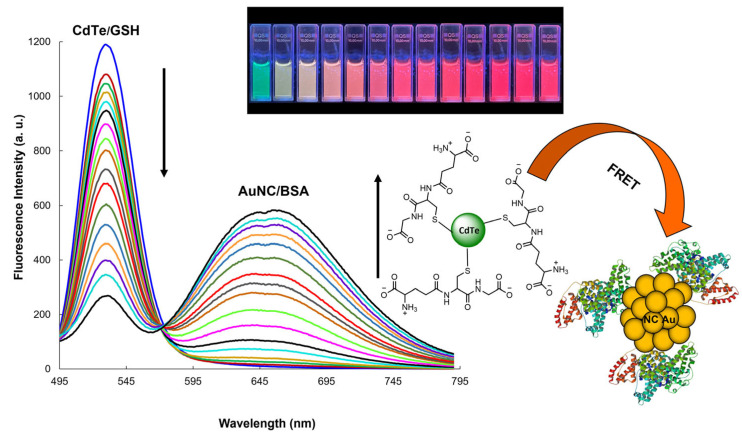
Fluorescence spectra of CdTe@GSH QDs in the presence of increasing concentrations of AuNCs@BSA (0–1.44 mg/mL). The colored curves indicate the decrease in donor emission at 530 nm (downward arrow) and the simultaneous increase in acceptor emission at 660 nm (upward arrow) due to FRET. The cuvette photograph illustrates the gradual color change under UV light as the AuNCs@BSA concentration increases.

The potential of CdTe/GSH QDs as fluorescent energy donors and AuNCs/BSA as energy acceptors in FRET assays was investigated. In the binding reaction between CdTe/GSH QDs and AuNCs/BSA, electrostatic interactions play a key role, leading to the formation of QDs/GSH-BSA/NCs complexes [33]. These interactions help bring the donor and acceptor into the necessary proximity for the FRET process to take place. A key factor influencing the efficiency of the FRET process is the overlap between the fluorescence emission spectrum of the donor and the absorption spectrum of the acceptor. Figure S4 illustrates the spectral overlap between the absorption spectrum of AuNCs and the emission spectrum of CdTe/GSH QDs. As shown, there is a significant overlap, which is essential for achieving optimal FRET efficiency. Figure 3 presents the emission spectra from the stepwise titration of CdTe/GSH QDs with AuNCs/BSA. The spectra show a gradual quenching of the donor fluorescence (CdTe/GSH QDs) and a corresponding enhancement of the acceptor fluorescence (AuNCs/BSA) as the concentration of AuNCs increases, indicating the occurrence of a FRET process. The main FRET parameters were determined as indicated in the Supplementary Information. Table 1 presents the overlap integral (*J*), FRET efficiency (*E*), Förster distance (*R*_0_), donor–acceptor distance (*r*), and the binding constant (*K*) for the CdTe/GSH QDs-AuNCs/BSA energy transfer assembly. The energy transfer was confirmed for the studied pair, as the *R_0_* and *r* values fall within the 2 to 8 nm range, and the condition 0.5*R*_0_ < *r* < 1.5*R*_0_ was met [34].

### 2.4. The Detection of DA by Using CdTe/GSH-AuNCs/BSA Assembly

The CdTe/GSH-AuNCs/BSA conjugate was used to detect DA in phosphate buffer (pH 8), showing notable changes in fluorescence intensity. Initially, the presence of DA facilitates interactions between the donor (CdTe/GSH QDs) and acceptor (AuNCs/BSA), resulting in a decrease in donor fluorescence at 530 nm and an increase in acceptor fluorescence at 660 nm, consistent with a FRET mechanism. However, with subsequent DA additions, the donor’s fluorescence signal undergoes complete quenching, while the acceptor’s signal does not increase further, indicating a significant non-FRET contribution that can be attributed to electron transfer (ET) from the CdTe/GSH QDs to DA.

**Figure 4:**
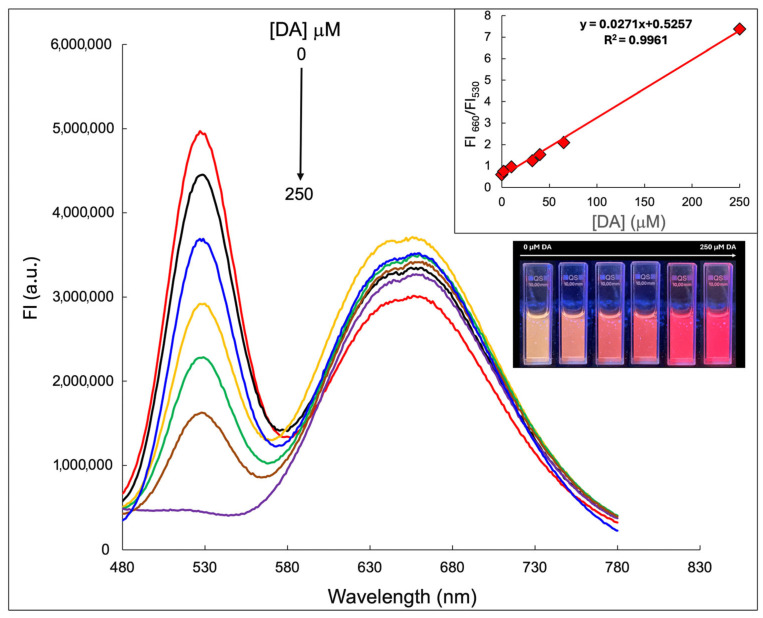
Fluorescence spectra of CdTe/GSH–AuNCs/BSA in the presence of DA. The colored curves represent the spectra recorded at increasing DA concentrations (0–250 µM). The cuvette photograph illustrates the color change under UV light with increasing DA concentration, and the inset shows the linear ratiometric calibration plot (FI_660_/FI_530_ vs. [DA]).

Figure 4 shows the fluorescence spectra of the donor–acceptor conjugate in absence and presence of DA. The fluorescence intensity ratio at 660 nm and 530 nm (FI_660_/FI_530_) exhibited a linear response to DA over a range of 0 to 250 μM (Inset, Figure 4), with a detection limit of 6.9 nM (determined as three times the standard deviation of the blank signal). These findings indicate that the CdTe/GSH-AuNCs/BSA conjugate functions as an excellent ratiometric nanosensor for sensitive DA detection.

Figure S5 shows the Stern Volmer graphic. It can be appreciated a linearity in a concentration range from 0 to 2.5 × 10^−4^ M, which indicates only one type of quenching (static or dynamic) in this concentration range. The Stern Volmer constant is 4.3 × 10^4^ M^−1^.

### 2.5. Response Mechanism of CdTe/GSH-AuNCs/BSA Assembly to DA

The quenching mechanism involves both FRET and ET processes. At low DA concentrations, DA mediates interactions between CdTe/GSH QDs (donor) and AuNCs/BSA (acceptor). DA interacts with BSA through hydrogen bonding and van der Waals forces [35], and with GSH through hydrogen bonding between the carboxyl groups of GSH-CdTe QDs and the amino groups of DA [36]. These interactions bring the donor and acceptor into the necessary proximity for the FRET process to occur. Consequently, a decrease in donor emission at 530 nm and an enhancement of acceptor emission at 660 nm are observed.

At higher DA concentrations, the donor fluorescence is completely quenched without further enhancement of the acceptor emission, indicating an additional non-FRET contribution. This behavior can be attributed to ET from CdTe/GSH QDs to oxidized DA. Under slightly alkaline conditions (pH 8), DA rapidly oxidizes to quinone species in the presence of ambient O_2_ (see Equation (S1) in the Supplementary Materials). These dopamine-quinone species are adsorbed onto the QD surface through strong non-covalent interactions, facilitating efficient ET from the excited QDs to DA and leading to fluorescence quenching. DA is a well-known quencher of QDs via non-radiative electron–hole recombination [37], acting as an efficient electron acceptor for QDs [38,39].

**Figure 5:**
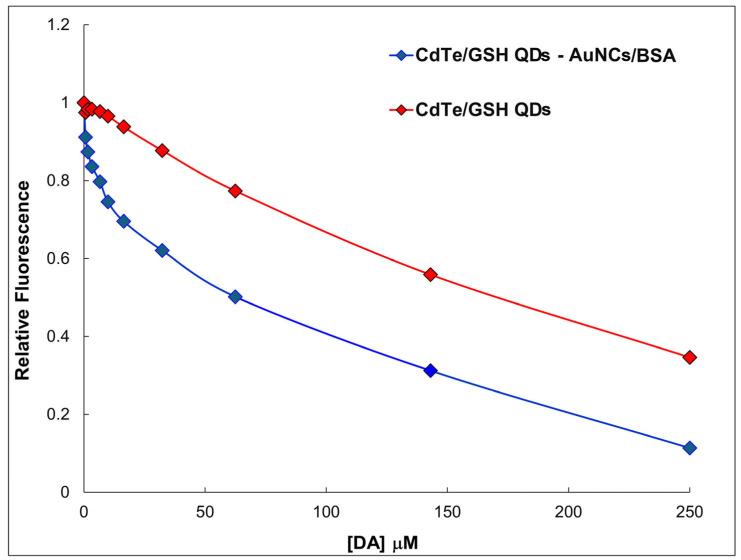
Response profile measured at 530 nm of CdTeQDs/GSH (red) and CdTe/GSH QDs-AuNC/BSA assembly (blue) toward DA.

To validate the proposed mechanism, Figure 5 presents the fluorescence response profiles at 530 nm for CdTe QDs/GSH (red) and the CdTe QDs/GSH-AuNCs/BSA assembly (blue) in the presence of DA. The profiles exhibit distinct behaviors, suggesting different response mechanisms. During initial titration, the assembly shows a steeper fluorescence response than CdTe/GSH QDs, reflecting the contribution of FRET. At higher DA concentrations, both systems converge to similar quenching behavior, confirming ET as the dominant pathway. In CdTe/GSH QDs, quenching proceeds exclusively via ET, whereas in the hybrid assembly it arises from the combined action of FRET and ET.

**Figure 6:**
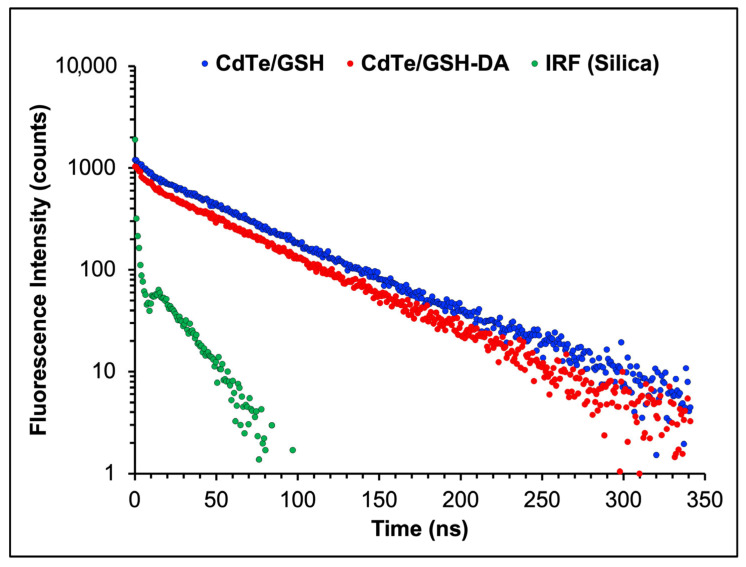
Fluorescence decay profiles of CdTe@GSH (blue) and CdTe@GSH–DA (red) with the experimental IRF (green). The shorter lifetime of CdTe@GSH–DA confirms fluorescence quenching by DA.

To gain insight into the quenching effect, we analyzed the optical behavior of CdTe/GSH QDs both with and without DA using time-resolved fluorescence spectroscopy (Figure 6).

A biexponential model with reconvolution of the experimental IRF was used to fit the fluorescence decay curves of CdTe@GSH and CdTe@GSH–DA. The corresponding fitting parameters (pre-exponential factors A_i_, lifetimes τ_i_, chi-square χ^2^ and intensity-weighted average lifetime ⟨τ⟩) are summarized in Table 2. The parameters τ_1_ and τ_2_ correspond to the fast and slow decay components, which reflect nonradiative surface-related processes and core-state recombination, respectively. The presence of DA introduces an additional quenching pathway that facilitates rapid electron transfer from the excited CdTe@GSH nanocrystals to DA molecules adsorbed on the surface. As a consequence, a reduction is observed in both τ_2_ and in the intensity-weighted average lifetime (⟨τ⟩), the latter decreasing from 38.4 ns for CdTe@GSH to 33.9 ns for CdTe@GSH–DA. This decrease in lifetime confirms the occurrence of photoinduced electron transfer and indicates that dopamine-quinone species bound to the QD surface act as efficient electron acceptors, promoting fluorescence quenching of the CdTe@GSH QDs. The nearly identical χ^2^ values (~1.0) obtained for both fits confirm the adequacy of the biexponential reconvolution model in describing the decay kinetics.

Zeta potential measurements of the QDs, both in the absence and presence of DA, were also conducted. The results showed that the zeta potential of CdTe/GSH QDs is −50.21 mV in the absence of DA and shifts to −29.63 mV after interaction with 30 µM DA, confirming that electrons are transferred from the QDs to DA.

The interaction of DA with AuNCs/BSA does not cause significant changes in the UV–visible absorption or fluorescence spectra (Figure S6), indicating that no new species are formed (the increasing band at 280 nm is attributed to DA) and that the fluorescence band of NCs at 660 nm remains unaffected in response to DA, consistent with the proposed mechanism.

Overall, DA quenches CdTe/GSH QDs through a dual mechanism: FRET dominates at low DA concentrations in the CdTe/GSH-AuNC/BSA assembly, while ET to dopamine-quinone species governs at higher concentrations. The combined spectroscopic, lifetime, and electrokinetic data validate this dual-mode quenching pathway.

### 2.6. Selectivity of CdTe/GSH-AuNCs/BSA Assembly for the DA Detection

**Figure 7:**
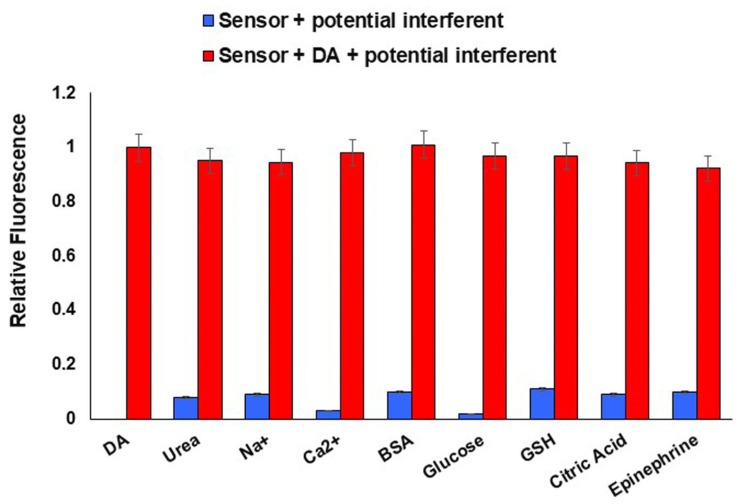
Relative fluorescence response (FI_660_/FI_530_) of CdTe/GSH-AuNCs/BSA + potential interferent (blue) and CdTe/GSH-AuNCs/BSA + DA + potential interferent (red). Experimental conditions: DA, 2.3 µM; potential interferent, 100 µM; pH 8.

The influence of potential interfering substances commonly found in different samples was investigated. Various representative substances, including urea, bovine serum albumin (BSA), glucose, glutathione (GSH), citric acid, epinephrine, and the metal ions Na^+^ and Ca^2+^, were analyzed at pH 8. Selectivity was evaluated through direct sensor assays in the presence of each potential interferent, as well as through competitive assays involving both DA and the potential interferent. The method’s selectivity arises from the specific properties of DA, which enable the donor–acceptor interactions required for FRET and the redox activity that supports ET. A DA concentration of 32.3 µM was selected for these experiments, corresponding to the conditions used in the standard addition method for real sample analysis. The standard addition method provided a calibration curve over the 0–65 µM range. This approach is applicable to various matrices, including urine, plasma, and pharmaceutical formulations, covering a broad range of DA concentrations. The results of these studies are presented in Figure 7. The fluorescence intensity ratio at 660 nm and 530 nm (FI_660_/FI_530_) was measured in the presence of 32.3 µM DA and 100 µM of each interference substance. The molar ratio of DA to interference was 1:3.

As shown in Figure 7, the relative fluorescence of CdTe/GSH–AuNCs/BSA sensor, both in direct assays in the presence of each potential interferent and in competitive assays involving each potential interferent and DA, showed no significant variations in the fluorescence signal (error below 10%), demonstrating the selective response of the nanosensor toward DA over other substances. Even at concentrations as high as 100 µM, these potential interferents did not affect the relative fluorescence intensity of the nanosensor. Therefore, the proposed nanosensor exhibits high selectivity for DA detection.

The presence of DA leads to a reduction in the fluorescent signal of the CdTe/GSH-AuNCs/BSA through a coordinated mechanism involving FRET and ET, both of which occur exclusively in the presence of DA. The sensor’s selectivity is based on these specific interactions between the nanosensor and DA.

### 2.7. Comparison with Other DA Sensors

The sensor was compared with other methods reported in the literature. Table 3 presents the linear range and detection limits of various nanostructure-based sensors for DA detection. Our sensor offers a broad linear range and a low detection limit, comparable to the lowest values reported, along with a reliable ratiometric signal. This method provides good sensitivity without requiring extensive off-line preparation or costly equipment.

### 2.8. The Ratiometric Determination of DA in Real Sample

A urine sample was collected from a 24-year-old male to confirm the applicability of this ratiometric sensor for DA determination. The proposed sensor was used to directly detect DA without prior sample treatment at pH 8 (phosphate buffer), following the standard addition method. Results from the sample analysis are presented in Table 4. Recovery tests were performed by adding four different DA concentrations to each sample. As shown in Table 4, there was good agreement between the added and measured amounts of DA in spiked urine samples, with recovery rates between 99% and 105% and a relative standard deviation (RSD) below 5%, demonstrating the reliability of this FRET fluorescent probe for DA detection. The concentration of DA in the analyzed sample falls within the reported range for a young male. The results represent the mean of three parallel analyses.

### 2.9. Screening Tests of Cytocompatibility

**Figure 8:**
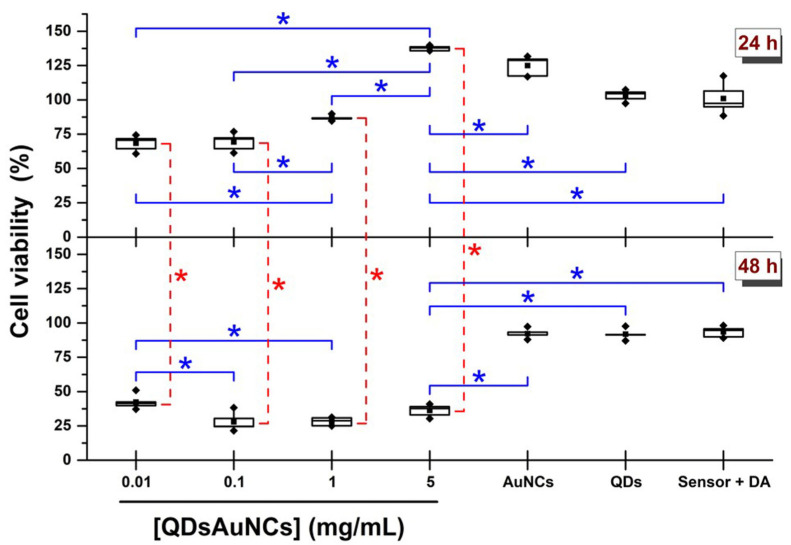
Viability of HeLa epithelial cells (cervical cancer cells) after exposure to the assemblies for 24 and 48 h. Individual AuNCs and CdTe QDs, as well as the sensor–DA conjugate, were included as controls for comparison with the individual samples. All samples were prepared and diluted in PBS. The final concentration of AuNCs, CdTe QDs, and the sensor–DA conjugate used as controls was 5 mg mL^−1^, expressed in terms of total solids. Statistically significant differences between data (*p* < 0.05) are denoted with *.

Figure 8 shows the cell viability of the assembly, their individual components, and the conjugate with DA upon incubation with HeLa epithelial cells (cervical cancer cells) for 24 and 48 h. At 24 h, all tested samples were found to display a cytocompatible response (yielding cell viability levels well above 70%, required to be deemed cytocompatible), and even eliciting an enhanced proliferative activity of the cultured cells when working at the highest concentration regime (5 mg/mL; with cell viability levels well above 100%). This high cytocompatibility and enhanced proliferative activity can be ascribed to the nature of the capping materials of both individual components of the assemblies. On the one hand, albumin (the capping material of the AuNCs) has been recognized to boost the activity of the Na^+^/H^+^ exchanger isoform 3 (NHE3) at intracellular levels of human epithelial cells [50], promoting their Na^+^ reabsorptive capacity and an improved concomitant proliferation (cell viabilities > 100%), with no change in levels of apoptosis [51]. On the other hand, glutathione (the capping material of the CdTe QDs) has been regarded as a promoter of proliferative responses in many normal and malignant cells, proving itself an essential element for the cell cycle progression [52]. In our opinion, the observed cytocompatibility and enhanced cell proliferation, combined with the exceptional photophysical properties described above, constitute an asset of the developed assemblies, paving the way for the exploration of additional activities of biological interest, such as intracellular sensing, labeling, and/or staining of both fixed and live cells, worth considering in upcoming research. In any case, the observed intrinsic cytocompatibility and enhanced cell proliferation, combined with the exceptional photophysical properties described above, constitute an asset of the developed assemblies, paving the way for the exploration of additional activities of biological interest, such as intracellular sensing, labeling, and/or staining of both fixed and live cells, worth considering in upcoming research. Regarding the samples incubated for 48 h, all of them, independently of their nature and concentration, showed a decrease in their cytocompatibility as compared to the time point of 24 h. The decrease in cytocompatibility of nanomaterials over time has been observed previously and is described as a multifactorial phenomenon, often tied to dynamic long-term interactions between the nanomaterial and the biological microenvironment [53,54,55].

## 3. Materials and Methods

### 3.1. Reagents and Apparatus

Cadmium chloride hemi(pentahydrate) (CdCl_2_·2.5H_2_O, 81%), potassium tellurite (K_2_TeO_3_, 90+%), glutathione (GSH, 98%), sodium borohydride (NaBH_4_, 99.99+%), sodium hydroxide (97%), ascorbic acid (AA, 99+%), citric acid (CA, 98+%), glucose (98+%), bovine serum albumin (BSA, 98%) and fluorescein (quantum yield QY, 79%) were purchased from Sigma-Aldrich, Mexico. Additional reagents (analytical grade) and solvents (spectroscopic grade) were sourced from Sigma-Aldrich (Naucalpan de Juárez, México).

Absorption spectra were recorded with a Shimadzu spectrophotometer UV-2700 (Shimadzu, Kyoto, Japan) with a 1 cm quartz cell. Emission spectra were carried out on a Horiba NanoLog fluorescence spectrophotometer (HORIBA Scientific, Edison, NJ, USA) using a xenon lamp as the excitation source and a 1 cm quartz cell. Micrographs were captured using a transmission electron microscope JEM-2200FS (JEOL, Akishima, Japan) set at an accelerating voltage of 200 kV and featuring spherical aberration correction in STEM mode. A high-angle annular dark field (HAADF) detector was used to obtain the images. Surface charge measurements were carried out using a STABINO ZETA Zeta Potential Analyzer (Microtrac MRB, Haan, Alemania). pH studies were measured with an AQUASEARCHER™ AB33PH pH meter (OHAUS, Ciudad de México, Mexico). Fluorescence lifetime assessments were conducted with an EasyLife™ X filter-based system (Horiba Scientific, Kyoto, Japan).

Dulbecco’s modified Eagle medium (DMEM), phosphate-buffered saline (PBS), glutaraldehyde solution (25 *w*/*v*% in water), orthophosphoric acid, formic acid, 2-N-morpholinoethanesulfonic acid (MES), and crystal violet were purchased from Sigma-Aldrich, (Naucalpan de Juárez, Mexico). Fetal bovine serum (FBS), MEM non-essential amino acids (NEAA; 100×), sodium pyruvate (100 mM), penicillin-streptomycin (pen-strep; 100 mg/mL), and trypsin-EDTA (0.25%, 0.913 mM EDTA) solutions were from Invitrogen. The 96-well plates and T-25 cell culture flasks were from Corning Costar (Glendale, AZ, USA). Epithelial HeLa cells (cervical cancer cells) were purchased from the American Type Culture Collection (ATCC), Manassas, VA, USA.

### 3.2. Preparation of BSA-Coated Au Nanocluster

The synthesis of BSA-coated fluorescent AuNCs was conducted via an aqueous solution method described in the literature [32] with slight modifications. In an amber flask, 5 mL of a 0.75 mM BSA solution and 5 mL of a 10 mM HAuCl_4_ solution were combined under orbital stirring at 200 rpm for 10 min. After that, 0.5 mL of NaOH (1 M) was introduced, and the mixture was incubated at 37 °C for 12 h with magnetic stirring at 50 rpm. The resulting AuNCs-BSA underwent dialysis for 24 h and were then lyophilized for 48 h.

### 3.3. Preparation of GSH-Coated CdTe QDs

GSH-coated CdTe quantum dots were synthesized according to the methodology reported in previous works of the group [56]. In this research, QDs with maximum emission wavelength at 557 nm were used for optimization and efficiency in FRET systems.

The relative quantum yields (QY) of CdTe/GSH QDs and AuNCs/BSA were determined as indicated in the Supplementary Information.

### 3.4. FRET Assays Between CdTe/GSH QDs and AuNCs/BSA

#### Selection of Optimal Condition to FRET Process

Donor CdTe/GSH QDs were selected based on the spectral overlap between their emission spectra and the absorption spectra of AuNCs/BSA. To determine the optimal excitation wavelength for the FRET process between CdTe/GSH QDs and AuNCs/BSA, the absorption spectrum of AuNCs/BSA was measured in the range of 200 to 700 nm. The most suitable excitation wavelength for the donor was found to be 400 nm, which is close to the minimal absorption of the acceptor.

FRET probes were performed using 3 mL of QDs solution (0.5 μM) and titrated by the successive addition of AuNCs/BSA stock solution under stirring condition. All solutions were prepared in phosphate buffer (pH 8). Resultant QDs-NCs conjugate emission spectra were recorded. All samples were excited at 400 nm.

### 3.5. Stability Study

The photochemical stability was assessed by exposing the nanomaterial to UV irradiation (365 nm) at a constant temperature using a 450 W xenon lamp. The material was dispersed in deionized water at pH 8 with a phosphate buffer (PBS). Fluorescence intensity was recorded for 60 min using the “kinetic” mode of the Nanolog spectrofluorometer.

### 3.6. Lifetime Measurements

Fluorescence lifetimes of CdTe/GSH and CdTe/GSH in the presence of DA were measured using an EasyLife X time-resolved spectrofluorometer (Horiba Scientific) equipped with a pulsed LED laser. For the CdTe/GSH QDs lifetime measurement, a sample was prepared under the specified conditions and placed in a quartz cuvette. The measurement was repeated after adding DA to the sample. Lifetime measurements were obtained by exciting the sample with a 490 nm laser source, and the resulting emission was collected at 528 nm. Ludox HS-40 colloidal silica served as the reference for the lifetime measurements.

### 3.7. Calibration Curve

The conjugate was prepared by mixing CdTe/GSH QDs with AuNCs/BSA solution to achieve an energy transfer efficiency of 50%, corresponding to an intensity ratio at the acceptor (660 nm) and donor (530 nm) maximum emission wavelengths of 1.5 (FI_660_/FI_530_ ≈ 1.5). Then, in 5 mL volumetric flasks, 2 mL of the conjugated QDs-NCs, 1 mL of PBS buffer solution (pH 8), and varying concentrations of DA (0.66 to 250 µM) were sequentially added and diluted to the final volume. The fluorescence spectra of the nanosensor were obtained in an emission range of 480 to 780 nm with an excitation wavelength of 400 nm.

### 3.8. Interference Study

The sensor’s response to other compounds was investigated using fluorescence spectroscopy. Interference assays were conducted for Na^+^ and Ca^2+^ ions, urea, BSA, glucose, GSH, and citric acid. Fluorescence intensity was measured in the presence of 32.3 µM DA and each interfering substance at a concentration of 100 µM, maintaining a DA-to-interference molar ratio of 1:3. For competition experiments, 3 mL of the conjugate solution was placed in a 1 cm path-length quartz fluorescence cuvette and mixed with a DA and coexisting substance solution at the same DA-to-interference molar ratio of 1:3. The fluorescence intensity ratio (FI_660_/FI_530_) was recorded in the presence of each potential interference.

### 3.9. Detection of DA in Real Samples

DA determination was conducted on a urine sample from a healthy 24-year-old adult using the standard addition method. The urine samples were analyzed directly for DA without any pretreatment. A 100 µL urine sample was mixed with the CdTeQDs-AuNCs conjugate in a buffer solution (pH 8). The conjugate was prepared by mixing CdTe/GSH QDs with AuNCs/BSA solution to achieve an energy transfer efficiency of 50%, corresponding to an intensity ratio at the acceptor (660 nm) and donor (530 nm) maximum emission wavelengths of 1.5 (FI_660_/FI_530_ ≈ 1.5). The standard addition method was applied for DA quantification, generating a standard addition curve over a concentration range of 0 to 65 µM. Results were based on three repetitions, yielding a high linearity (R^2^ = 0.9974) with the calibration equation y = 40,438x + 728,560.

### 3.10. Cell Culture Conditions

HeLa cells were cultured in DMEM medium supplemented with 10% FBS, 0.1 mM NEAA, 1 mM sodium pyruvate, and 1% penicillin-streptomycin, and maintained under standard conditions (37 °C, 5% CO_2_ in a humidified atmosphere) in T-25 cell culture flasks. The cells were either passaged or seeded for experiments once they reached 80–90% confluence. To achieve this, confluent monolayers were treated with trypsin-EDTA solution and incubated for 4 min under culture conditions to facilitate detachment. The cells were then collected by centrifugation, resuspended in fresh culture medium, and either passaged or seeded as required. All procedures were conducted under sterile conditions, using sterile-filtered Milli-Q water.

### 3.11. Screening Test for Cytocompatibility

The screening cytocompatibility tests were conducted in accordance with the ISO 10993-5:2009 international standard [57], using HeLa epithelial cells (cervical cancer cells) as the testing line and the crystal violet colorimetric assay as the testing method. Cytocompatibility was assessed using the crystal violet assay [58,59,60,61,62,63]. HeLa cells were chosen for this experiment because of their highly proliferative response, which helps prevent or compensate for misleading cytotoxicity outcomes (false positive or negative results) [64,65], known to occur and arguably overestimated when employing cell lines with low-to-moderate proliferation rates (such as senescent cells, specialized lines, or mature neurons where dopamine plays essential roles, to cite a few) [66,67]. Likewise, the crystal violet assay was preferred over other standard methods, such as MTT, MTS, XTT, and LDH, given the presence of metal species, albumin, and glutathione in our samples, which have been demonstrated to interfere with the formazan production and/or enzymatic activity of these standard tests [66,67,68]. Briefly, the conjugates were prepared as described in Section 3.4, by mixing 15 µL of CdTe–GSH QDs with 20 µL of AuNCs in 2 mL of PBS buffer. The dispersions were lyophilized in batch mode to obtain powders corresponding to a final concentration of 5 mg mL^−1^ in total solids. For cytocompatibility assays, the lyophilized conjugates, together with AuNCs/BSA and CdTe/GSH QDs alone, were reconstituted in 1× PBS to prepare stock solutions of 5 mg mL^−1^, which were then serially diluted as required. HeLa cells were seeded in 96-well plates (100 µL, 1.5 × 10^4^ cells/well) the day before the experiment and cultured under standard conditions for 24 h. On the day of the experiment, the medium was replaced by the sample solutions (100 µL/well), and cells were incubated for 24 or 48 h prior to the viability test.

After the incubation periods, the culture medium was discarded, and the cells were incubated at room temperature (300 rpm, 15 min) with 10 µL of glutaraldehyde solution (11 *w*/*v*% in water). The solution was discarded, and the cells were washed twice with Milli-Q water. Next, the cells were incubated at room temperature (300 rpm, 15 min) with 100 µL of crystal violet solution (0.1 *w*/*v*% in a mixture of 200 mM orthophosphoric acid, 200 mM formic acid, and 200 mM MES, pH 6). The solution was discarded, and the cells were washed twice with Milli-Q water. After washing, the cells were allowed to dry overnight at room temperature. Once dried, the cells were incubated at room temperature (300 rpm, 15 min) with 100 µL of acetic acid solution (10 w/w% in water). The absorbance of the resulting solution was measured at 595 nm. The percentage of cell viability was quantified as

(1) $$Cellviability=100*\frac{A_{S}}{A_{C}}$$

where *A_S_* and *A_C_* represent the absorbance of the treated cells (exposed to the samples) and control cells (exposed to 1× PBS), respectively. The data presented are the averages from five independent experiments. Sterile-filtered Milli-Q water was used throughout the process, and all experiments were conducted under sterile conditions.

### 3.12. Statistical Analysis

The statistical analysis was conducted using two-way analysis of variance (ANOVA). Post hoc Bonferroni and Tukey tests were applied for multiple comparisons, with differences deemed significant at a *p*-value of less than 0.05.

## 4. Conclusions

A ratiometric fluorescent sensor was developed based on the assembly between the green-emitting CdTe/GSH QDs and the red-emitting AuNCs/BSA. The presence of DA leads to a decrease in the donor’s fluorescence signal and an increase in the acceptor’s fluorescence signal, consistent with a FRET mechanism. Subsequent additions of DA lead to a complete quenching of the donor’s fluorescence signal, without any increase in the acceptor’s signal, indicating a significant non-FRET contribution, which is attributed to ET. The nanosensor exhibits high selectivity for DA over other potential interfering molecules, with a detection limit of 6.9 nM. Its effectiveness was confirmed through successful DA detection in urine samples. Additionally, the nanosensor demonstrates excellent cytocompatibility and promotes cell proliferation in HeLa epithelial cells, making it suitable for other biologically relevant applications, such as intracellular sensing and live cell staining for microscopy. This material shows significant potential for use in both environmental and biological fields.

## Figures and Tables

**Table 1:** FRET parameters of the CdTe/GSH QDs-AuNCs/BSA conjugate.

Donor (D)	Acceptor (A)	*J* (cm^3^Lmol^−1^)	*E*	*R*_0_ (nm)	*r* (nm)	*K* (M^−1^)
CdTe/GSH	AuNCs/BSA	5.9 × 10^−13^	0.78	5.9	4.8	8.4 × 10^3^

**Table 2:** Fluorescence lifetime parameters of CdTe@GSH and CdTe@GSH–DA (30 μM) systems obtained from reconvolution fitting.

Sample	τ_1_ (ns)	A_1_ (%)	τ_2_ (ns)	A_2_ (%)	χ^2^	⟨τ⟩ (ns)
CdTe/GSH	11.39 ± 0.63	17.8	50.56 ± 0.81	82.2	0.967	38.4
QDs/GSH-DA	9.52 ± 0.53	18.7	46.02 ± 0.74	81.3	0.967	33.9

**Table 3:** Comparison of different nanomaterials-based fluorescence sensors for the determination of DA.

Sensor	Lineal Range	Detection Limit	Reference
NCs Au/BSA	1–10 µM	10 nM	[40]
CdSe/ZnS-DNA	2 × 10^−5^ –1 × 10^−4^ M	29.3 nM	[41]
Carbon Dots (CDs)	0.1 × 10^−3^–0.5 × 10^−3^ M	33 µM	[42]
CdTe/TGA	0.1–0.3 × 10^−3^ M	6 × 10^−3^ M	[43]
CDs	0.3–100 µM	93 nM	[44]
B,S co-doped graphene QDs	0–340 μM	3.6 μM	[45]
Silica coated QDs	0.0005–0.1 mM	2.41 × 10^−4^ mM	[37]
N-doped CDs	2–20 μg/mL	1.97 μg/mL	[46]
Carbon dots FRET	1–50 μM	20 nM	[47]
CDs-AuNCs FRET	5–180 nM	2.9 nM	[48]
CdS/thioglycolic acid	3.94 × 10^−7^–4.67 × 10^−8^ M	2.55 × 10^−9^ M	[49]
CdTe/GSH-NCsAu/BSA FRET	0–250 µM	6.9 nM	(this work)

**Table 4:** Recoveries of DA in urine samples (*n* = 3).

Sample	Found (µM)	Added (µM)	Found (µM)	Recovery (%)
Urine	1.8	3.3	5.3	103.5
		9.9	12.2	104.2
		32.3	34.9	102.3
		62.5	63.7	99.1

## Data Availability

Data are contained within the article.

## Supplementary Materials

The following supporting information can be downloaded at: https://www.mdpi.com/article/10.3390/molecules30214169/s1. Figure S1 (a) Evolution of absorption and fluorescence spectra of CdTe/GSH QDs at different reaction times. (b) CdTe/GSH QDS under UV light. Table S1 Optical properties of CdTe@GSH quantum dots. Figure S2 Optical properties of CdTe@GSH quantum dots. Figure S3 Stability of CdTe/GSH QDs (blue) and AuNCs/BSA (red). Figure S4 Overlapping of the CdTe/GSH QDs emission spectrum and AuNCs/BSA absorption spectrum. Figure S5 Stern Volmer plot for the FRET assembly between QD/GSH and NCsAu/BSA. Figure S6 (a) UV Vis and (b) fluorescence spectra of AuNCs/BSA in the presence of increasing amounts of DA. Oxidation of dopamine (Equation (1)). The link to consult the procedure for determining quantum yields and the calculation of the main parameters of the FRET process is indicated in the supplementary materials.
