# Supplementary material for: Cytocompatible FRET Assembly of CdTe@GSH Quantum Dots and Au@BSA Nanoclusters: A Novel Ratiometric Strategy for Dopamine Detection
Source: Molecules. 2025 Oct 23;30(21):4169. doi: 10.3390/molecules30214169 (PMC12610238; doi:10.3390/molecules30214169)
Supplement: Supplementary file 1 [file molecules-30-04169-s001.zip › molecules-3876138-supplementary.pdf]

SUPLEMENTARY MATERIALS

CYTOCOMPATIBLE FRET ASSEMBLY OF CdTe@GSH QUANTUM DOTS AND Au@BSA NANOCLUSTERS: A NOVEL RATIOMETRIC STRATEGY FOR DOPAMINE DETECTION

Arturo Iván Pavón-Hernández[a], Doris Ramírez-Herrera[a], Eustolia Rodríguez-Velázquez[b], Manuel Alatorre-Meda[c], Miguel Ramos-Heredia[a], Antonio Tirado-Guízar[a], Georgina Pina-Luis[a*]

[a]*Centro de Graduados e Investigación en Química, Tecnológico Nacional de México/Instituto Tecnológico de Tijuana, Tijuana 22500, BC, México*

[b]*Facultad de Odontología, Universidad Autónoma de Baja California, Calzada Universidad 14418, Parque Industrial Internacional, Tijuana 22390, BC, Mexico*

[c]*Cátedras CONACyT-Tecnológico Nacional de México/I. T. Tijuana, Centro de Graduados e Investigación en Química-Grupo de Biomateriales y Nanomedicina, Blvd. Alberto Limón Padilla S/N, Tijuana 22510, BC, Mexico*

* Corresponding author at: Centro de Graduados e Investigación, Instituto Tecnológico de Tijuana, A.P. 1166, Tijuana 22500, BC, México.

Tel.: 52 664 6233772; 52 664 6234043.

E-mail address: gpinaluis@tectijuana.mx, gpinaluis@yahoo.com

a)

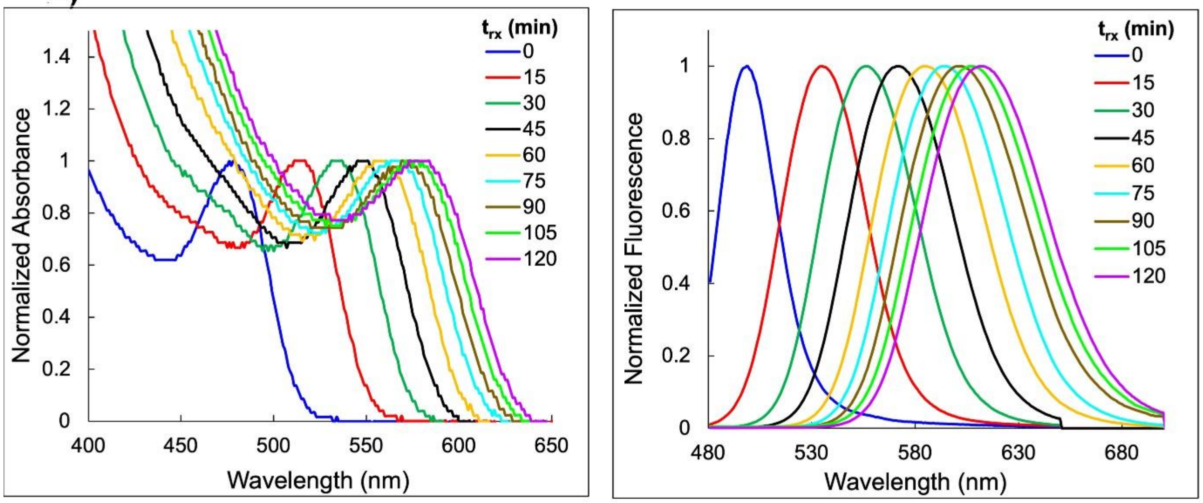

b)

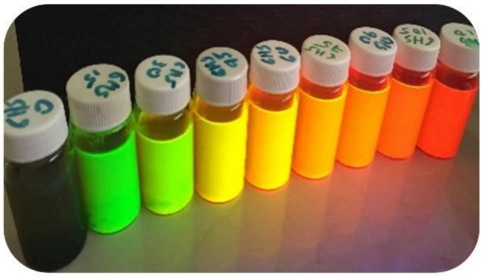

Figure S1. a) Evolution of absorption and fluorescence spectra of CdTe/GSH QDs at different reaction times. b) CdTe/GSH QDS under UV light.

Table S1. Optical properties of CdTe@GSH quantum dots

	t_{rx} (min)	λ_{ab} (nm)	D (nm)	λ_{em} (nm)	ε M^{-1} x cm^{-1}	% QY
**CdTe/GSH QDs**	0	477	1.57	498	26131.88	17
	15	513	2.67	535	80550.33	39
	30	535	3.05	557	106801.93	41
	45	548	3.23	571	120607.08	36
	60	558	3.32	585	127842.77	41
	75	565	3.39	595	133624.72	49
	90	571	3.43	602	136989.41	53
	105	576	3.47	607	140398.33	48
	120	580	3.50	612	142984.09	38

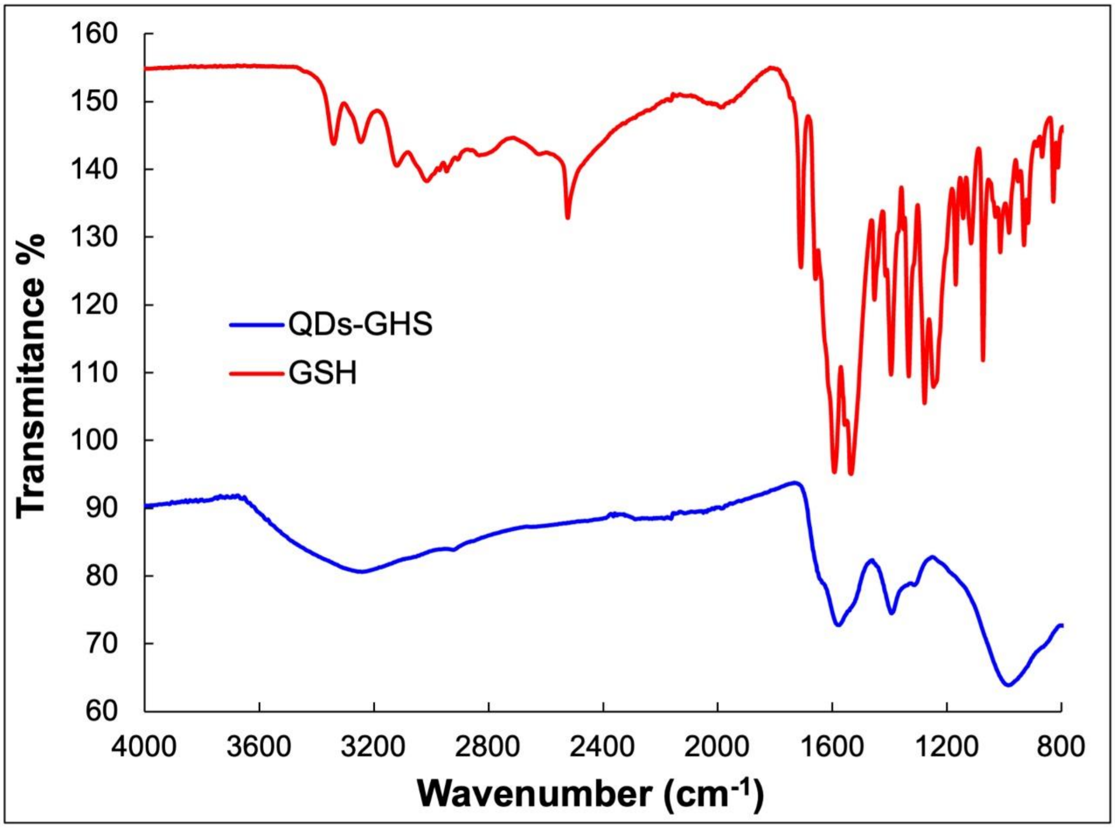

Figure S2. FT-IR spectra of glutathione (GSH) and CdTe/GSH QDs.

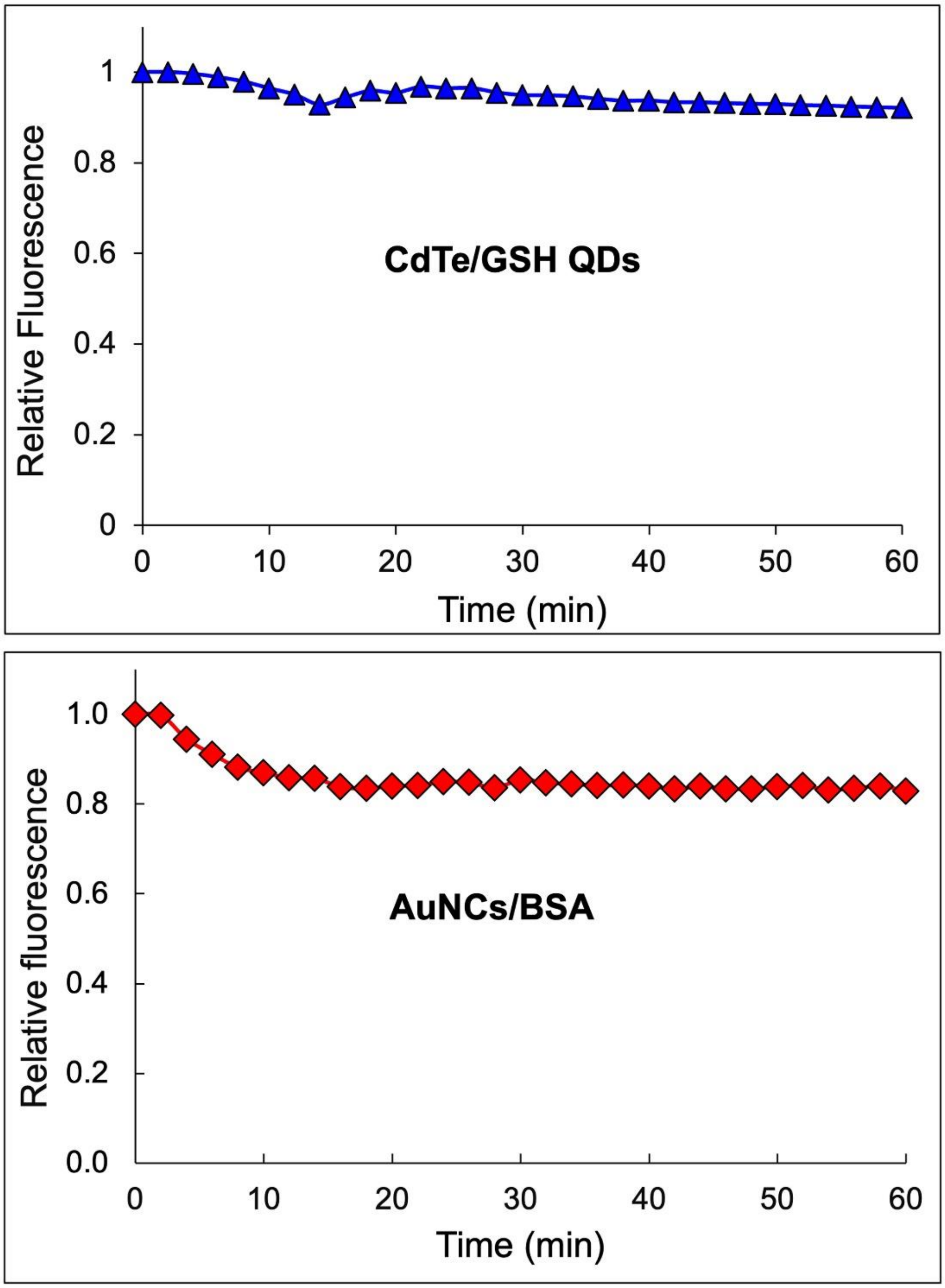

Figure S3. Stability of CdTe/GSH QDs (blue) and AuNCs/BSA (red).

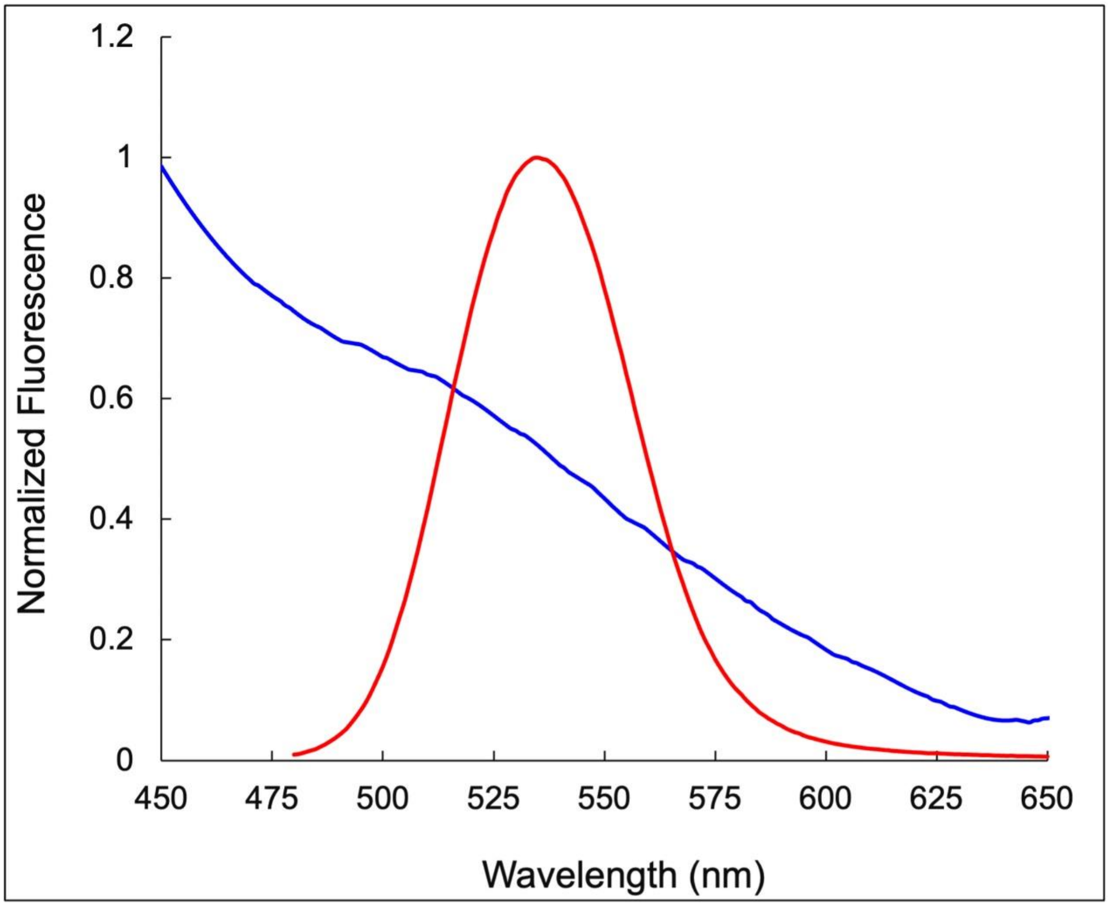

Figure S4. Overlapping of the CdTe/GSH QDs emission spectrum and AuNCs/BSA absorption spectrum.

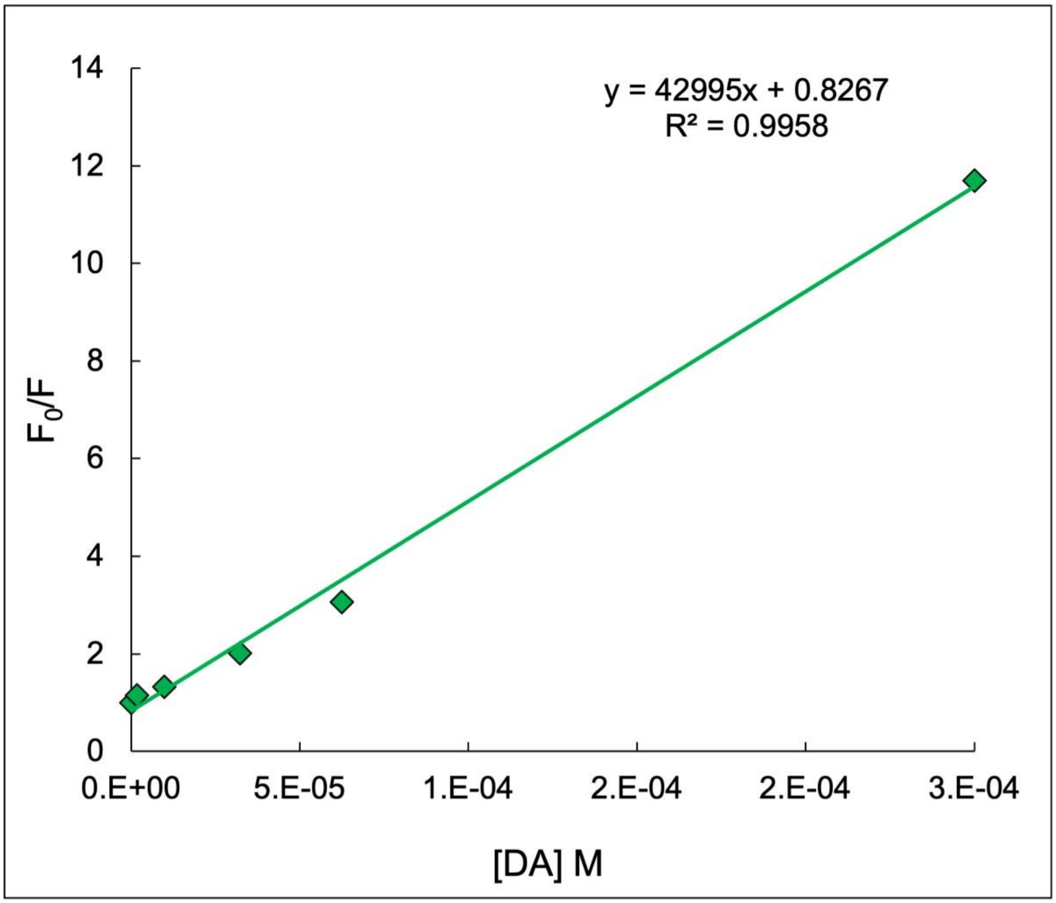

Figure S5. Stern Volmer plot for the FRET assembly between QD/GSH and NCsAu/BSA

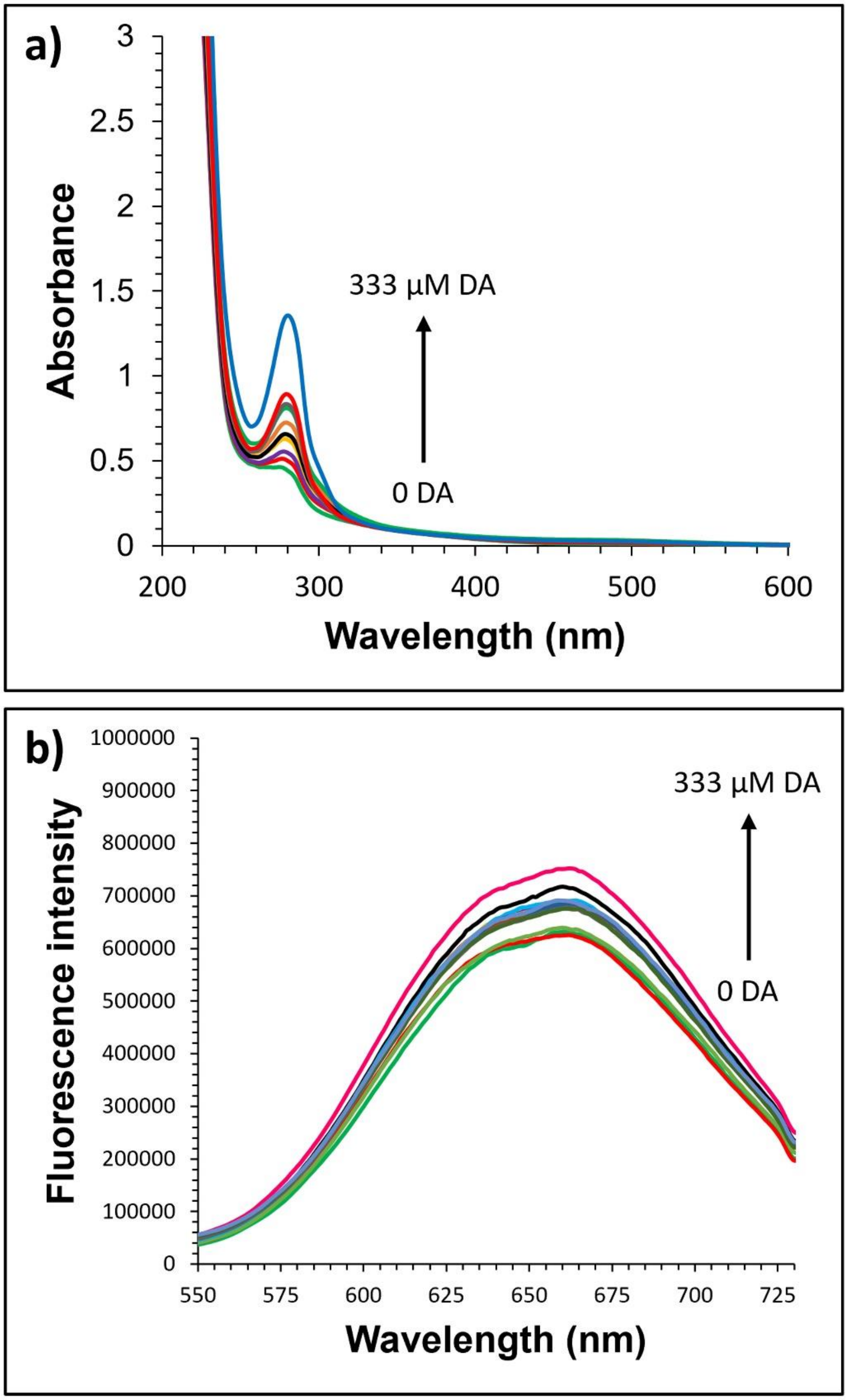

Figure S6. a) UV Vis and b) fluorescence spectra of AuNCs/BSA in the presence of increasing amounts of DA.

Oxidation of Dopamine

DA is oxidized through a reversible process involving 2 protons and 2 electrons, producing o-benzoquinone (dopamine-quinone), according to:

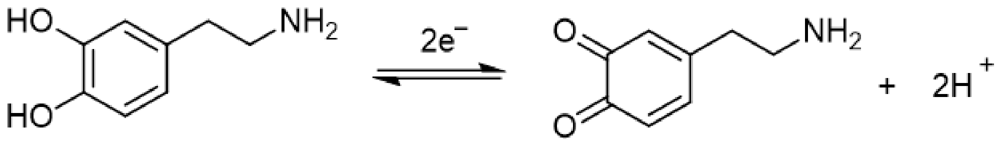

(S1)

Determination of the nanoparticles quantum yields

The relative quantum yields (QY) of CdTe/GSH QDs and AuNCs/BSA were determined as described in Nanomaterials 2021, 11, 1981. https://doi.org/10.3390/nano11081981

Calculation of the main parameters of the FRET process

The main FRET parameters were determined as described in Nanomaterials 2021, 11, 1981. https://doi.org/10.3390/nano11081981
